# Drought Induced Changes in Growth, Osmolyte Accumulation and Antioxidant Metabolism of Three Maize Hybrids

**DOI:** 10.3389/fpls.2017.00069

**Published:** 2017-02-06

**Authors:** Shakeel A. Anjum, Umair Ashraf, Mohsin Tanveer, Imran Khan, Saddam Hussain, Babar Shahzad, Ali Zohaib, Farhat Abbas, Muhammad F. Saleem, Iftikhar Ali, Long C. Wang

**Affiliations:** ^1^College of Agronomy and Biotechnology, Southwest UniversityChongqing, China; ^2^Department of Agronomy, University of AgricultureFaisalabad, Pakistan; ^3^Department of Crop Science and Technology, College of Agriculture, South China Agricultural UniversityGuangzhou, China; ^4^Scientific Observing and Experimental Station of Crop cultivation in South China, Ministry of AgricultureGuangzhou, China; ^5^School of Land and Food, University of TasmaniaHobart, TAS, Australia; ^6^College of Plant Science and Technology, Huazhong Agricultural UniversityWuhan, China; ^7^The Research Center for Ornamental Plants, College of Horticulture, South China Agricultural UniversityGuangzhou, China

**Keywords:** antioxidant defense, agronomic traits, drought, maize, ROS, yield formation

## Abstract

Consequences of drought stress in crop production systems are perhaps more deleterious than other abiotic stresses under changing climatic scenarios. Regulations of physio-biochemical responses of plants under drought stress can be used as markers for drought stress tolerance in selection and breeding. The present study was conducted to appraise the performance of three different maize hybrids (Dong Dan 80, Wan Dan 13, and Run Nong 35) under well-watered, low, moderate and SD conditions maintained at 100, 80, 60, and 40% of field capacity, respectively. Compared with well-watered conditions, drought stress caused oxidative stress by excessive production of reactive oxygen species (ROS) which led to reduced growth and yield formation in all maize hybrids; nevertheless, negative effects of drought stress were more prominent in Run Nong 35. Drought-induced osmolyte accumulation and strong enzymatic and non-enzymatic defense systems prevented the severe damage in Dong Dan 80. Overall performance of all maize hybrids under drought stress was recorded as: Dong Dan 80 > Wan Dan 13 > Run Nong 35 with 6.39, 7.35, and 16.55% yield reductions. Consequently, these biochemical traits and differential physiological responses might be helpful to develop drought tolerance genotypes that can withstand water-deficit conditions with minimum yield losses.

## Introduction

Drought stress imposes drastic effects on plant growth and development, agronomic traits and yield formation by altering physio-anatomical mechanisms. It disturbs plant-water relations, photosynthetic gas exchange capacities, cell turgor, source-sink relationships and various metabolic events in plants (Anjum et al., 2011b). Drought-induced production of reactive oxygen species (ROS) in terms of superoxide anions (O_2_^.-^), singlet oxygen ($O_{2}^{1}$), hydroxyl radicals (OH^-^), hydrogen peroxide (H_2_O_2_) and alkoxy radicals (RO) harms the cell membranes and damages the proteins, lipids and nucleic acids ultimately leading to cell death (Munné-Bosch and Penuelas, 2003).

Osmotic adjustment is an innate behavior of plants which helps them in maintaining water balance by synthesizing different osmolytes/solutes. These solutes protect cellular structures and functions as well as maintain water balance and delay dehydrative damage by maintaining cell turgor and other physiological mechanisms under water-deficit conditions (Taiz and Zeiger, 2006). Osmolytes further improve the carbohydrate partitioning during reproductive stages of the plants and improve final yield (Subbarao et al., 2000). Generally, proline accumulation increases under stress conditions, which not only helps in maintaining cell turgor but is also involved in quenching free radicals, maintaining sub-cellular structures, and buffering cellular redox potential (Ashraf and Foolad, 2007). Previously, increased protein contents, SS, total carbohydrates, and phenolic contents were also reported in maize plants under water-limited conditions (Anjum et al., 2011a,b,c). Anjum et al. (2014) also reported that lipids and proteins are the main target sites of oxidative stress in plants exposed to abiotic stresses. Furthermore, Maksup et al. (2014) also found enhanced protein accumulation in tolerant rice cultivars under water stress conditions.

Plants also possess an efficient antioxidant (enzymatic and non-enzymatic) defense system to cope with ROS-induced oxidative stress (Anjum et al., 2011b,c; Ashraf et al., 2015). Both enzymatic, i.e., APX, SOD, peroxidase (POD), catalase (CAT), DHAR, and MDHAR as well as non-enzymatic, i.e., AsA, DHA, GSH, GSSG antioxidants minimize the oxidative damage under stressful conditions. The contribution of both enzymatic and non-enzymatic antioxidants may ensure the stress tolerance in plants subjected to a long-term drought stress (Sharma et al., 2012). These antioxidants have been reported to contribute directly or indirectly in drought tolerance of maize. For instance, Adebayo and Menkir (2015) stated that sustained yields in maize under drought stress were directly related to better antioxidant activities. Farooq et al. (2009) also concluded that increased activities/levels of enzymatic and non-enzymatic antioxidants may improve the drought tolerance by scavenging ROS.

Maize (*Zea mays* L.) is well-recognized as one of the most important cereals worldwide while China ranked second in its production and consumption after USA (Gale et al., 2014). Normally, it needs 500–800 mm of water during its life cycle (80–110 days) (Critchley and Klaus, 1991); however, occurrence of drought stress during maize growth period may hamper the nitrogen and water use efficiencies leading to significant yield losses (Saini and Westgate, 2000; Ashraf et al., 2016). Drought-related physiological and metabolic changes might be helpful in determining the sensitivity or tolerance of a plant under water deficit conditions and can be used as stress indicators. For the selection and screening of drought-tolerant genotypes as well as for agronomic and genetic engineering, the expression of tolerance mechanisms and identification of most effective antioxidants in plants must be studied in detail at different drought levels (Xing and Wu, 2012). Therefore, the present study was conducted to assess the drought-induced oxidative damage in terms of ROS accumulation, and possible protection by osmoregulation and/or activation of enzymatic and non-enzymatic antioxidative defense systems. Involvement of these physio-biochemical mechanisms in maize growth and yield response under drought stress were also studied to get better insights of maize tolerance mechanism(s) to drought stress conditions.

## Materials and Methods

### Plant Material and Growth Conditions

A pot experiment was conducted in a glass house at the College of Agronomy and Biotechnology, Southwest University, Chongqing, China (latitude 29° 49′ 32′′ N, longitude 106° 26′ 02′′ E and altitude 220 m) during spring, 2013. The seeds of three maize hybrids, i.e., Dong Dan 80, Wan Dan 13, and Run Nong 35 were obtained from Liaoning Dongya Seed Company Ltd., Liaoning, China. Seeds of all maize hybrids were sown in PVC nursery trays (two seeds per hill). Two week old seedlings were transplanted (two seedlings per pot) into plastic pots (34 cm in diameter, 24 cm in depth) filled with sandy loam soil and farmyard manure in 3:1 proportion. Total weight of each pot was 16 kg after filling with air-dried soil. The experimental soil contained 2.08 g kg^-1^ total nitrogen, 3.77 g kg^-1^ total phosphorous, 12.33 g kg^-1^ total potassium, 89.37 mg kg^-1^ alkali-hydro nitrogen content, 30.14 mg kg^-1^ available phosphorous, 54.88 mg kg^-1^ available potassium, 14.76 g kg^-1^ organic matter and 6.48 pH. Fertilizer was applied to all pots at 15 g per pot (5 g at planting, 5 g 20 DAP and 5 g at 40 DAP), containing 15-5-5% N, P_2_O_5_ and K_2_O, respectively. The average night and day temperature of the glass house was in the range of 21–33°C during crop growth period, while the relative humidity (RH) was 52–88% in the morning and 49–83% in the afternoon.

### Drought Treatments

The maize plants were allowed to grow under normal conditions up to pre-tasseling stage. At 45 DAP, three different levels of drought stress with respect to FC, i.e., LD (80% FC), MRD (60% FC) and SD (40% FC) were imposed, while a well-watered control (Ck) with 100% FC was maintained for comparison. The stress treatments were regularly monitored by a moisture meter TRIME-EZ/-IT (IMKO Micromodultechnik GmbH, Germany) while the specified drought treatments were applied until crop maturity. The treatments were arranged in a completely randomized design (CRD) under factorial arrangement. Each treatment was replicated thrice, and there were five pots per replicate.

### Biochemical Assays

Healthy, undamaged and fully expanded plant leaves (third from the top) from each replication were sampled at 7 days after imposition of drought treatments to assess the osmolytes, ROS, and enzymatic and non-enzymatic antioxidants. After washing with double distilled water, leaves were frozen in liquid N_2_ and stored at -80°C until biochemical analyses.

#### ROS Production

The production rate of superoxide ion (O_2_^-^) was determined according to Elstner and Heupel (1976). Briefly, fresh leaf samples (0.5 g) were homogenized with 65 mM phosphate buffer (pH 7.8) and centrifuged at 5,000 × *g* for 15 min at 4°C. A mixture of 2 ml containing phosphate buffer (0.9 ml), 10 mM hydroxylamine hydrochloride (0.1 ml) and supernatant (1 ml) was incubated at 25°C for 30 min, then 1 ml of 17 mM sulphanilamide and 7 mM α-naphthylamine were added and incubated at 25°C for 20 min. The change in absorbance was measured at 530 nm. A standard curve with NO_2_^-^ was used to calculate the production rate of O_2_^-^ from the chemical reaction of O_2_^-^ and hydroxylamine. The H_2_O_2_ content was assayed according to the method described by Mukherjee and Choudhuri (1983).

#### Lipid Peroxidation Rate and Lipoxygenase (LOX) Activity

Malenoaldehyde was measured spectro-photometrically using the thiobarbituric (TBA) method according to Dhindsa et al. (1981). An aliquot of enzyme solution (2 ml) was added to a tube containing 1ml 20% trichloroacetic acid (TCA) and 0.5% TBA. The mixture was heated in a water bath at 95°C for 30 min, cooled to room temperature and then centrifuged at 14,000 rmp for 10 min. The absorbance was read at 532 nm and non-specific absorbance at 600 nm was subtracted from it. The MDA content was calculated by using an extinction coefficient of 155 mM^-1^ cm^-1^.

Lipoxygenase (LOX) activity was measured according to Minguez-Mosquera et al. (1993). The absorbance was read at 234 nm and the activity of LOX was expressed as nmol min^-1^ mg^-1^ protein. Thiobarbituric acid reactive substances (TBARS) in leaf tissues were evaluated as described by Cakmak and Horst (1991). Briefly, fresh leaf samples (0.5 g) were homogenized in 4 ml of 1% TCA and then1 ml of supernatant was mixed with 3 mL of 0.5% TBA in 20% TCA. The vials were closed tightly and placed in a water bath at 95°C for 2 h. To stop the reaction, vials were cooled down in an ice bath. The absorbance was read at 532 and 660 nm. Membrane permeability was determined by assessing the electrolyte leakage (EL) following the method of Lutts et al. (1996). The EL was calculated as: EL = (EC_1_/EC_2_) × 100.

#### Osmolyte Accumulation Profiles

Free proline (FP) content was assessed in fresh leaf samples (0.5 g) following the method of Bates et al. (1973) using ninhydrin. The reaction mixture was extracted with 5 ml toluene, cooled to room temperature and absorbance was read at 520 nm. TC were estimated by the phenol-sulphuric acid method as devised by Dubois et al. (1956). The absorbance was read at 485 nm against diluted sulphuric acid (5 + 2, *v/v*) at the same wavelength. Total carbohydrate contents were calculated by measuring the difference between these two readings. SS were estimated by the anthrone-sulphuric method following Fales (1951). Fresh leaves (0.5 g) were put into 15 ml distilled water and boiled in a water bath for 20 min. After cooling, 5 ml anthrone was added to 0.1 ml of boiled sample. The 3 ml of boiled sample was transferred to a cuvette and the absorbance was read at 620 nm. The TPC was estimated following a slightly modified method of Ainsworth and Gillespie (2007) by using Folin-Ciocalteu reagent. The reaction mixture was composed of 0.1 ml extract, 7.9 ml distilled water, 0.5 ml Folin–Ciocalteu reagent, and 1.5 ml of 20% sodium carbonate. The absorbance of the resulting mixtures was read at 750 nm and TPC were measured from a standard curve developed by gallic acid (GA) standards. SP content was estimated by the Bradford (1976) method. Bradford solution (1 ml) was mixed with 100 μl crude extract and absorbance was read at 595 nm. The protein contents were estimated from a standard curve. TFA were assessed by using the ninhydrin colorimetric method of Huang et al. (2010). The absorbance was read at 568 nm.

#### Antioxidant Activity

##### Enzymatic antioxidants

Superoxide dismutase (EC 1.15.1.1) activity was assayed using the kit (A001-1) provided by Nanjing Jiancheng Bioengineering Institute, China. One unit of SOD activity was defined as the amount of enzyme required for 1 mg tissue proteins in 1 ml of a reaction mixture to raise SOD inhibition rates to 50% at 550 nm (Tecan infinite M200, Swit). Peroxidase (POD, EC1.11.1.7) activity was determined following the method of Upadhyaya et al. (1985). The absorbance of the reaction mixture containing 100 μl enzyme extract, 50 mM phosphate buffer (pH 7.0), 28 μl guaiacol and 19 μl H_2_O_2_ was read at 420 with a 30 s interval up to 2 min and used the absorbance change 0.01 as a POD activity. Catalase (CAT, EC 1.11.1.6) activity was determined by using the kit (A007-1) purchased from Nanjing Jiancheng Bioengineering Institute, China. One unit of CAT activity was estimated as the amount of enzyme that decomposes 1 μmol H_2_O_2_ at 405 nm sec^-1^ in 1 mg fresh tissue proteins (Tecan infinite M200, Swit). APX (EC 1.11.1.11) was assayed according to Nakano and Asada (1981). The reaction mixture (3 ml) contained 100 μl enzyme extract, 100 mM phosphate buffer (pH 7.0), 0.3 mM ascorbic acid, 0.1 mM EDTA-Na2, and 0.06 mM H_2_O_2_. The change in absorbance after adding H_2_O_2_ was read at 290 nm for 2 min at every 30 s interval. MDHAR (EC 1.6.5.4) was assayed by following the method of Foyer et al. (1989). The enzyme activity was assayed by following the change in wavelength at 340 nm after adding ascorbate oxidase whereas DHAR (EC 1.8.5.1) was assayed by following the method described by Doulis et al. (1997). A 1 ml reaction mixture contained 50 μl of enzyme extract, 1 ml of 50 mM potassium phosphate buffer (pH 7.0), 0.2 mM DHA, 2.5 mM GSH, 1, and 0.1 mM EDTA. The absorbance was read at 265 nm.

##### Non-enzymatic antioxidants

Total ascorbate (AsA + DHA) and reduced ascorbate (AsA) contents were estimated according to Hodges et al. (1996). A 200 μl of supernatant was mixed with 500 μl of 150 mM K_2_PO_4_ buffer (pH 7.0) containing 5 mM EDTA and 100 μl of 10 mM dithiothreitol to reduce DHA to AsA. After 15 min, 100 μl of 0.5% N-ethylmaleimide was added to the reaction mixture at 25°C to remove extra dichlorodiphenyltrichloroethane (DDT). To determine AsA, 200 μl deionized water was used instead of DTT and N-ethylmaleimide. Color appeared in both mixtures with the addition of 400 μl of 44% o-phosphoric acid, 400 μl of 10% trichloro acetic acid (TCA), 200 μl of 30 g l^-1^ FeCl_3_ and 400 μl of a,a’-dipyridyl in 70% (*v/v*) ethanol. The solutions were placed at 40°C for 1 h and absorbance was read at 525 nm. Ascorbate contents were estimated from a standard curve whereas the amount of DHA was calculated from the difference between the total (AsA + DHA) and reduced ascorbate (AsA). Total glutathione (GSH + GSSG) and glutathione disulphide (GSSG) contents were measured by the method of Anderson (1985). The amount of GSH was calculated from the difference between the total glutathione and GSSG.

### Growth and Yield

Leaf area of maize plants was recorded with leaf area meter (Li-Cor 3100, Li-Cor, Lincoln, NE, USA) while a meter scale and an electronic weighing balance were used to measure plant height and biomass accumulation, respectively. Before recording the plant dry biomass, harvested plants were cut into pieces and kept in an oven at 80°C until constant weight. To determine growth and yield related attributes, 30 plants (10 plants from each replicate) were sampled randomly and harvested at maturity. The harvested plants were sun-dried (in an open place) and the ears were shelled manually to record GY per plant. The total plant dry biomass was weighed for each treatment and regarded as BY. To record 100-kernel weight, three random samples of 100-kernels were taken from the seed lot of each treatment, weighed and averaged. Harvest index (HI) was calculated as the percent ratio of GY and BY.

### Statistical Analysis

The data collected were statistically analyzed following the analysis of variance technique using SPSS 16.0 (SPSS, Chicago, IL, USA) software whilst the differences amongst treatments were separated according to Newman–Keuls tests at a significance level of 5%. SigmaPlot 9.0 (Systat Software Inc., San Jose, CA, USA) was used for graphical presentation of the data.

## Results

### Variations in Agronomic Traits and Maize Yield under Drought Stress

Drought stress severely inhibited the agronomic traits and yield of maize, however, all three maize hybrids showed differential response to drought stress. Compared with well-watered control, SD stress (40% FC) significantly reduced the leaf area, shoot fresh and dry weights, number of leaves/plant, kernel rows/ear, kernels/ear, 100-grain weight, GY and BY of all three maize hybrids (**Tables 1** and **2**). Although, a decreasing trend was observed with increase in drought stress levels in all the tested hybrids, nonetheless, drought-induced adversities were more prominent in Run Nong 35 than those in Dong Dan 80 or Wan Dan 13 (**Table 1** and **2**).

**Table 1:** Agronomic traits of three maize hybrids as influenced by different drought stress levels.

Maize hybrids	Treatments	Plant height (cm)	Leaf area (cm^2^)	Shoot fresh weight/plant (g)	Shoot dry weight/plant (g)	Number of leaves/plant
Dong Dan 80	Ck	202.45 ± 3.76^a^	245.56 ± 2.32^a^	275.01 ± 6.34^a^	72.54 ± 2.06^a^	14.88 ± 0.33^a^
	LD	201.87 ± 4.56^a^	243.04 ± 3.23^a^	267.23 ± 6.43^ab^	68.67 ± 2.05^a^	14.68 ± 0.32^ab^
	MRD	198.67 ± 3.78^a^	240.43 ± 3.76^ab^	260.89 ± 4.89^b^	67.34 ± 2.34^a^	14.22 ± 0.23^b^
	SD	195.45 ± 5.98^a^	237.23 ± 1.45^b^	251.87 ± 1.45^b^	62.78 ± 1.15^b^	13.87 ± 0.16^b^
	Means	199.61	241.565	263.75	67.83	14.41
Wan Dan 13	Ck	201.65 ± 1.89^a^	244.94 ± 2.21^a^	272.67 ± 4.78^a^	72.78 ± 1.32^a^	14.34 ± 0.19^a^
	LD	199.34 ± 5.87^ab^	241.32 ± 2.54^ab^	265.4 ± 5.54^ab^	65.87 ± 0.99^b^	14.21 ± 0.21^a^
	MRD	196.56 ± 2.89^b^	236.06 ± 3.12^b^	257.67 ± 5.11^b^	64.67 ± 1.77^bc^	13.67 ± 0.21^b^
	SD	192.89 ± 4.78^b^	230.67 ± 2.76^b^	247.67 ± 2.76^b^	60.67 ± 1.78^c^	12.78 ± 0.31^c^
	Means	197.61	238.25	260.85	66.00	13.75
Run Nong 35	Ck	201.65 ± 2.22^a^	243.66 ± 2.65^a^	273.56 ± 5.21^a^	71.45 ± 1.57^a^	13.89 ± 0.14^a^
	LD	198.65 ± 3.54^a^	237.11 ± 1.87^b^	263.34 ± 4.81^a^	64.34 ± 1.46^b^	13.11 ± 0.13^b^
	MRD	192.56 ± 1.87^b^	230.23 ± 2.87^c^	241.21 ± 3.54^b^	58.89 ± 0.88^c^	10.89 ± 0.29^c^
	SD	181.67 ± 3.55^c^	220.43 ± 3.23^d^	224.98 ± 3.23^c^	49.98 ± 0.67^d^	9.54 ± 0.14^d^
	Means	193.63	232.86	250.77	61.17	11.86

*Values are means of three replicates ± SE. Values followed by the similar lower case letters within columns for a maize hybrid don’t differ significantly according to Newman–Keuls test (*P* < 0.05).Ck, control (100% FC); LD, low drought (80% FC); MRD, moderate drought (60% FC); and SD, severe drought (40% FC); FC, Field capacity*.

**Table 2:** Yield and related characteristics of three maize hybrids as influenced by different drought stress levels.

Maize cultivars	Treatments	Ears/plant	Kernel rows/ear	Kernels/ear	100-kernel weight (g)	Grain yield/plant (g)	Biological yield/plant (g)
Dong Dan 80	Ck	1.22 ± 0.02^a^	14.78 ± 0.24^a^	472.35 ± 3.43^a^	23.98 ± 0.22^a^	138.56 ± 0.89^a^	331.77 ± 3.34^a^
	LD	1.20 ± 0.06^a^	14.68 ± 0.19^a^	469.46 ± 5.76^a^	23.09 ± 0.65^ab^	129.21 ± 1.67^b^	329.78 ± 4.23^a^
	MRD	1.19 ± 0.09^a^	14.04 ± 0.38^b^	466.13 ± 4.34^a^	22.78 ± 0.22^b^	126.45 ± 1.67^b^	319.78 ± 3.76^b^
	SD	1.15 ± 0.06^a^	13.65 ± 0.41^b^	459.26 ± 2.87^b^	21.56 ± 0.25^c^	119.65 ± 0.98^c^	310.56 ± 4.87^c^
	Means	1.19	14.29	466.80	22.85	128.47.22	322.97
Wan Dan 13	Ck	1.21 ± 0.05^a^	14.21 ± 0.31^a^	474.10 ± 2.87^a^	22.87 ± 0.35^a^	134.67 ± 1.21^a^	327.99 ± 2.65^a^
	LD	1.19 ± 0.08^a^	14.08 ± 0.23^a^	465.90 ± 2.78^b^	21.78 ± 0.34^b^	126.76 ± 0.76^b^	324.76 ± 2.86^a^
	MRD	1.17 ± 0.05^a^	13.67 ± 0.16^b^	459.06 ± 4.76^b^	20.67 ± 0.35^c^	118.65 ± 1.21^c^	315.67 ± 5.87^b^
	SD	1.13 ± 0.08^a^	12.32 ± 0.32^c^	447.14 ± 6.09^c^	19.65 ± 0.21^d^	104.34 ± 1.55^d^	303.89 ± 3.76^c^
	Means	1.18	13.57	461.55	21.24	121.11	318.08
Run Nong 35	Ck	1.21 ± 0.03^a^	14.08 ± 0.32^a^	471.44 ± 2.87^a^	23.07 ± 0.13^a^	133.76 ± 1.34^a^	328.67 ± 4.11^a^
	LD	1.17 ± 0.05^ab^	13.79 ± 0.21^a^	456.33 ± 4.33^b^	21.11 ± 0.21^b^	116.11 ± 1.22^b^	315.67 ± 3.76^b^
	MRD	1.14 ± 0.04^ab^	11.67 ± 0.26^b^	423.47 ± 2.67^c^	19.55 ± 0.18^c^	93.65 ± 1.79^c^	303.78 ± 6.12^c^
	SD	1.10 ± 0.03^b^	9.67 ± 0.21^c^	380.12 ± 3.65^d^	17.45 ± 0.22^d^	76.12 ± 1.11^d^	274.27 ± 4.21^d^
	Means	1.16	12.30	432.84	20.30s	104.91	305.60

*Values are means of three replicates ± SE. Values followed by the similar lower case letters within columns for a maize hybrid don’t differ significantly according to Newman–Keuls test (*P* < 0.05). Ck, control (100% FC); LD, low drought (80% FC); MRD, moderate drought (60% FC); and SD, severe drought (40% FC). FC, Field capacity*.

### ROS Production and LOX Activity in Maize Hybrids Response to Drought Stress

**FIGURE 1:**
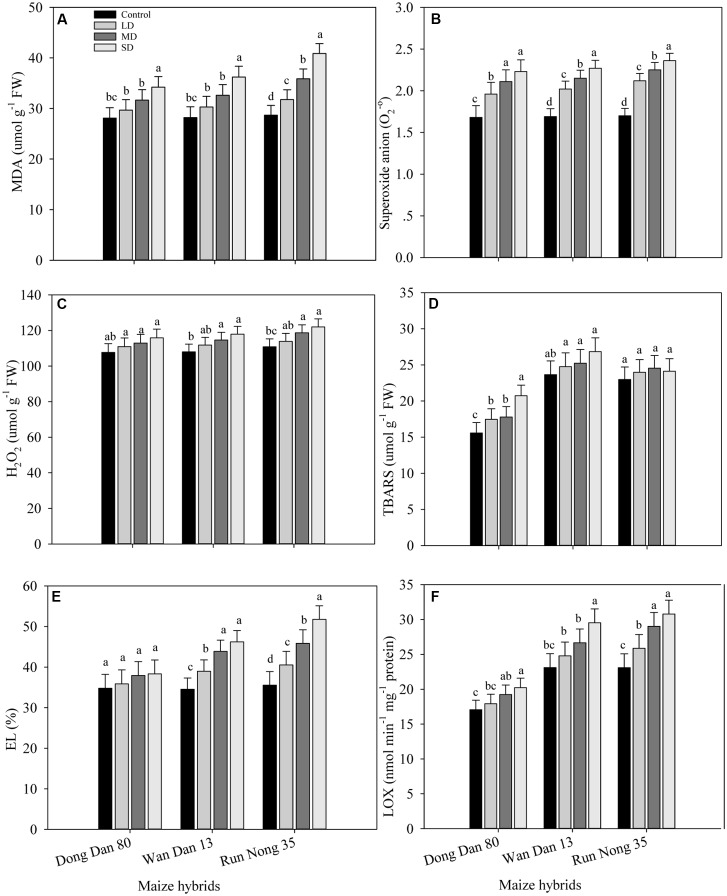
**Influence of different drought stress levels on the production of (A)** malenoaldehyde (MDA), **(B)** superoxide anion (O_2_^.-^), **(C)** hydrogen peroxide (H_2_O_2_), **(D)** thiobarbituric acid reactive substances (TBARS), **(E)** electrolyte leakage (EL), and **(F)** lipoxygenase (LOX) in three maize hybrids. Capped bars above means represent ±SE of three replicates. Small alphabetical letters above means denote the significant differences among treatment with in a maize hybrid at *P* ≤ 0.05. Ck, control (100% FC); LD, low drought (80% FC); MRD, moderate drought (60% FC); and SD, severe drought (40% FC); FC, Field capacity.

The levels of ROS accumulation and membrane damage in all maize hybrids were increased under drought stress. When compared with well-watered control, drought stress treatments increased the values of MDA, O_2_^-^, H_2_O_2_, TBARS, EL, and LOX in the range of 10–46% in Run Nong 35, 9–34% in Wan Dan 13, and 5–24% in Dong Dan 80, respectively. Overall, oxidative stress in terms of ROS production was increased with increased drought levels with more severe oxidative stress at maximum level of drought stress (40% FC). Additionally, low ROS activity and less membrane damage were recorded in Dong Dan 80 followed by Wan Dan 13. However, the maximum oxidative damage in Run Nong 35 indicated its sensitivity to drought stress (**Figures 1A–F**).

### Osmolytes Accumulation in Maize Hybrids in Response to Drought Stress

**FIGURE 2:**
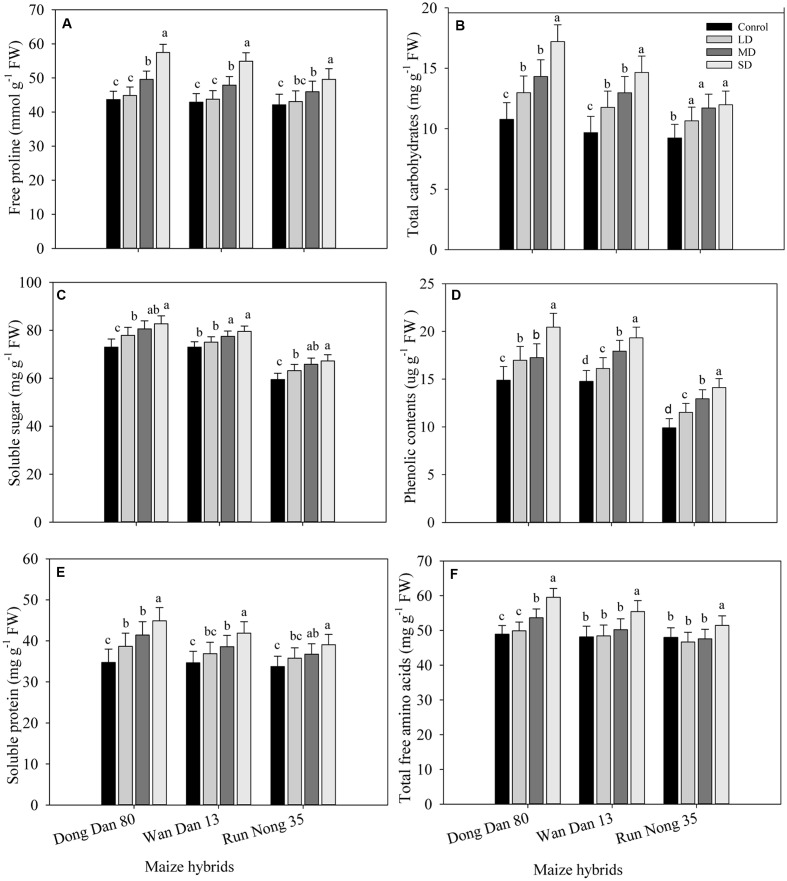
**Influence of different drought stress levels on the accumulations of (A)** free proline, **(B)** total carbohydrates, **(C)** soluble sugars, **(D)** phenolic contents, **(E)** soluble protein, and **(F)** total free amino acids in three maize hybrids. Capped bars above means represent ±SE of three replicates. Small alphabetical letters above means denote the significant differences among treatment with in a maize hybrid at *P* ≤ 0.05. Ck, control (100% FC); LD, low drought (80% FC); MRD, moderate drought (60% FC); and SD, severe drought (40% FC); FC, Field capacity.

Drought stress triggered the production and accumulation of different osmolytes in all maize hybrids. Concentrations of proline, carbohydrates, SS, phenolics, proteins, and TFA were considerably higher under SD conditions as compared to well-watered control. Accumulations of these osmolytes in all maize hybrids were increased with the severity of drought stress (SD > MRD > LD > Ck). Variations regarding osmolytes accumulation were also apparent among maize hybrids; the maximum accumulations were recorded in Dong Dan 80 followed by Wan Dan 13 and Run Nong 35, which indicated that Dong Dan 80 may perform better under water deficit conditions (**Figures 2A–F**).

### Activities of Enzymatic Antioxidants in Maize Hybrids under Drought Stress

**FIGURE 3:**
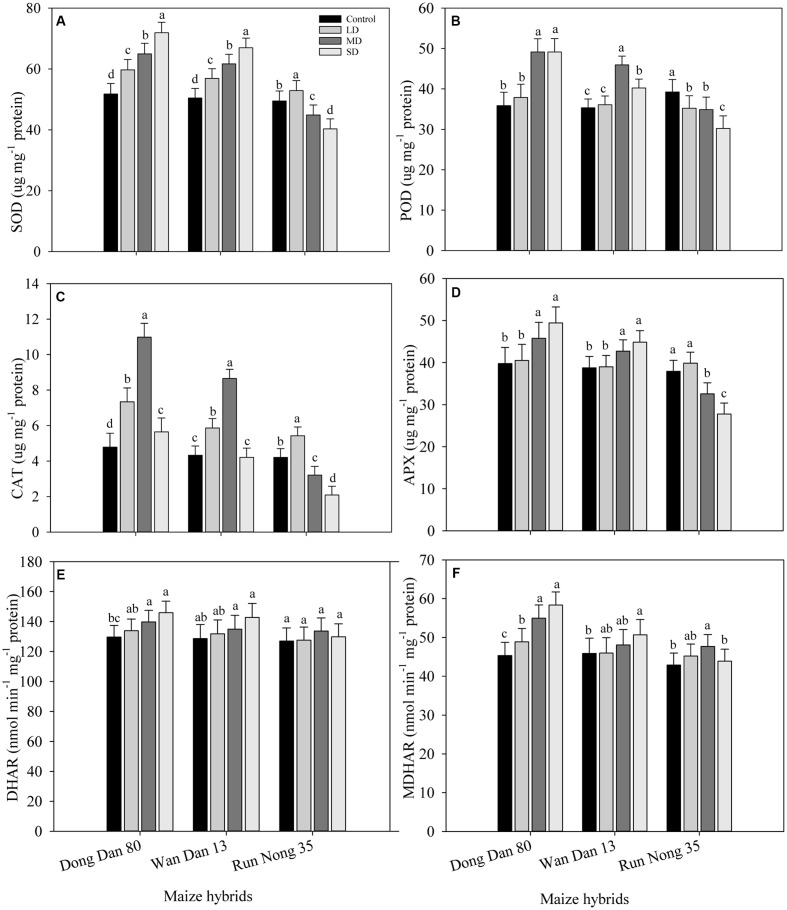
**Influence of different drought stress levels on the activities of (A)** superoxide dismutase (SOD), **(B)** peroxidase (POD), **(C)** catalase (CAT), **(D)** Ascorbate peroxidase (APX), **(E)** Dehydroascorbater eductase (DHAR), and **(F)** monodehydroascorbater eductase (MDHAR) in three maize hybrids. Capped bars above means represent ±SE of three replicates. Small alphabetical letters above means denote the significant differences among treatment with in a maize hybrid at *P* ≤ 0.05. Ck, control (100% FC); LD, low drought (80% FC); MRD, moderate drought (60% FC); and SD, severe drought (40% FC); FC, Field capacity.

Responses of enzymatic antioxidants varied significantly (*P* < 0.05) under the influence of drought stress. Compared with well-watered control, activities of SOD in Don Dang 80 and Wan Dan 13 were significantly increased with the severity of drought stress; the maximum values were recorded at SD stress (40% FC). However, the activities of SOD in Run Nong 35 were dramatically reduced at SD stress, and the maximum values for SOD were recorded at LD stress level (80% FC) (**Figure 3A**). The maximum activities of POD and CAT in Don Dang 80 and Wan Dan 13 were recorded at MRD stress (60% FC), While in Run Nong 35, POD and CAT activities were maximum in well-watered control and LD stress (80% FC), respectively. The activities of POD and CAT varied among maize hybrids and followed the trend of Don Dang 80 > Wan Dan 13 > Run Nong 35 (**Figures 3B,C**). Moreover, patterns of APX, MDHAR, and DHAR activities were parallel to drought stress level (except APX in Run Nong 35), generally showing a linear increase with an increase in drought stress level. Activities of APX, MDHAR, and DHAR were increased by 24, 13, and 29% in Don Dang 80 and 16, 11, and 10% in Wan Dan 13 under SD conditions (40% FC), respectively. Contrarily, for Run Nong 35, activities of these three antioxidants were declined at SD; the maximum APX activity was observed at LD, while the maximum MDHAR and DHAR activities were recorded at MRD level (**Figures 3D–F**). Overall, Don Dang 80 showed greater activities of enzymatic antioxidants compared with Wan Dan 13 or Run Nong 35 (**Figures 3A–F**).

### Levels of Non-enzymatic Antioxidants in Maize Hybrids under Drought Stress

**FIGURE 4:**
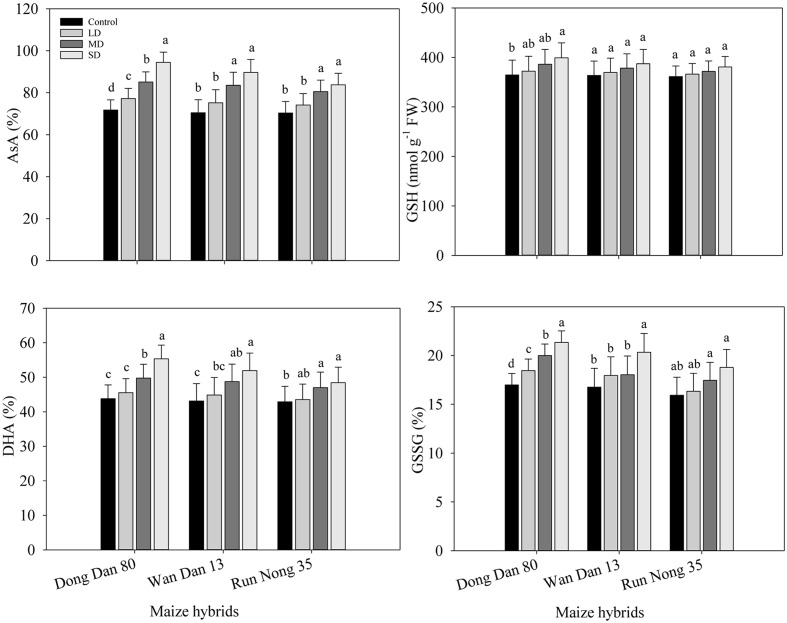
**Influence of different drought stress levels on (A)** ascorbic acid (AsA), **(B)** reduced glutathione (GSH), **(C)** dehydroascorbate (DHA), **(D)** oxidized glutathione (GSSG) in three maize hybrids. Small alphabetical letters above means denote the significant differences among treatment with in a maize hybrid at *P* ≤ 0.05. Capped bars above means represent ±SE of three replicates. Ck, control (100% FC); LD, low drought (80% FC); MRD, moderate drought (60% FC); and SD, severe drought (40% FC); FC, Field capacity.

Drought stress induced the changes in non-enzymatic antioxidants which led to different responses of three maize hybrids to water deficit conditions. Moderate and SD stress (60 and 40% FC) resulted in significantly higher AsA and DHA contents in all the maize hybrids. GSH contents were generally unaffected by drought stress in all maize hybrids except for Dong Dan 80, where GSH contents were significantly increased at 40% FC. Drought stress at any levels significantly increased the GSSG contents in Dong Dan 80, while did not significantly alter the GSSG in Run Nong 35. In Wan Dan 13, GSSG contents were only increased at SD stress to a significant level. Averaged across different drought stress levels, Dong Dan 80 and Run Nong 35 showed the maximum and minimum levels of non-enzymatic antioxidants, respectively (**Figures 4A–H**).

## Discussion

Drought is one of the major constraints for higher growth and productivity of field crops. Drought-induced adversities in plants demands the studies on exploring the drought tolerance mechanisms in plants to overcome significant yield losses under water stress (Singh et al., 2014, 2015). In this study, we accessed the osmolyte accumulation, antioxidant defense system and ROS-based variations in growth and yield performance of maize under drought stress. Drought stress severely inhibited growth, yield and related characteristics of maize, however, all three maize hybrids showed different responses in this regard. A decreasing trend was observed regarding maize performance with increase in drought stress levels, nonetheless, such drought-induced adversities were more prominent in Run Nong 35 than Dong Dan 80 or Wan Dan 13 (**Tables 1** and **2**). Previously in Chickpea, Mafakheri et al. (2010) noted reduced growth and severe yield losses when drought was imposed at reproductive stages. Moreover, Ge et al. (2012) also reported the reduced nutrient uptake, biomass accumulation and harvest index in maize under drought stress.

In the present study, ROS accumulation and membrane damage seemed to be higher under drought stress in all maize hybrids; however, effects were more apparent in Run Nong 35 which might be due to its sensitivity to drought stress (**Figures 1A–F**). Synthesis and accumulation of ROS is exaggerated under stressed conditions and aggressively damages the biological membranes and organic molecules. Moreover, enhanced lipid peroxidation in terms of MDA accumulation serves as an index of oxidative damage caused by ROS. Recently, Yanling et al. (2015) reported that enhanced anti-oxidant activities to quench ROS were related to drought tolerance in Chinese domesticated water melons (*C. lanatus var. lanatus*). Furthermore, drought stress caused an increase in EL compared to well-watered conditions in *Cassia occidentalis* (Srivastava and Srivastava, 2015). The drought tolerance in young oil palm plants was related to efficient protective mechanisms against ROS by activating enzymatic and non-enzymatic antioxidant strategies simultaneously (Silva et al., 2015).

Drought stress triggered the production and accumulation of different osmolytes in all maize hybrids. Concentrations of proline, carbohydrates, SS, phenolic contents, proteins, and TFA were considerably higher in all maize hybrids under SD conditions as compared to control; however, Dong Dan 80 accumulated more osmolytes than Wan Dan 13 or Run Nong 35 (**Figures 2A–F**). Accumulation of different compatible solutes and their involvement in osmotic adjustment, maintenance of cell turgor and protection of different cell structures might lead to significant improvement in drought tolerance in maize. It has been observed that moderate to SD affects the biosynthesis and accumulation of proline and soluble carbohydrates (Ghaderi and Siosemardeh, 2011). A significant increase in carbohydrate metabolites especially sugars and starches indicated a diurnal turnover under limited water supply in *Phoebe zhennan* plants, suggesting their availability to be metabolized in source organs or their translocation toward roots (Hu et al., 2015). The accumulation of some compatible solutes, i.e., proline and other free amino acids increased significantly in *Salicornia brachiata* under PEG-induced water stress that played dynamic roles in osmotic regulation, pH maintenance, protection of cellular macromolecules, and scavenging of free radicals to negate water stress (Parida and Jha, 2013). SD up-regulates the concentrations of free amino acids and SS that might be due to increased proteolysis under water-deficit conditions (Good and Maclagan, 1993; Alizadeh et al., 2011). The significant accumulation of phenolic compounds in tissues of drought tolerant plants and their powerful ROS scavenging roles indicate their roles against the oxidative damage caused by drought and salt stress (Reginato et al., 2015). Some previous reports also confirmed the contributions of different osmolytes in inducing drought tolerance in various crops (Tan et al., 2006; Farooq et al., 2009; Pawar et al., 2015).

In the present study, drought stress up-regulated the activities of anti-oxidative defense systems in all maize hybrids. Results showed that drought stress stimulated the enzymatic antioxidative defense system; however, activities of some antioxidants such as POD and CAT were reduced with severity of drought. Furthermore, all maize hybrids showed a variable response to drought stress, the activities of all antioxidants were generally higher in Dong Dan 80 than Wan Dan 13 or Run Nong 35. The enzymatic activities in Run Nong 35 were increased at initial drought levels but decreased dramatically at SD levels, exhibiting drought sensitive behavior of Run Nong 35 (**Figures 3A–F**).

The contents of AsA, GSH, GSSG, DHA and their combined concentrations were higher at higher levels of drought; the concentrations of these non-enzymatic antioxidants were higher in Dong Dan 80 than those in Wan Dan 13 or Run Nong 35. Drought stress induced changes in non-enzymatic antioxidants that led to different responses of three maize hybrids to water deficit conditions (**Figures 4A–H**). Previous studies proved that higher activities/levels of enzymatic and non-enzymatic antioxidants are important to induce drought tolerance. For example, a better anti-oxidative defense system may provide protection against oxidative stress and enhance plant tolerance under drought conditions. A key role of antioxidants in drought tolerance has also been reported in various crops including rice (Yang et al., 2014), sugarcane (Sales et al., 2015), and wheat (Kaur and Zhawar, 2015). Furthermore, enzymes involved in the ascorbate-glutathione cycle, i.e., APX, DHAR, and MDHAR are also important to enhance drought tolerance of a plant by quenching superoxide radicals and H_2_O_2_ (Fazeli et al., 2007). The AsA, GSH, α-tocopherol and carotenoids might be good indicators for drought stress tolerance (Guha et al., 2012). In this study, involvement of AsA against ROS formation under drought stress cannot be ignored; however, on the other hand, glutathione metabolism and pools of GSH are directly or indirectly related to the responses of plants against various environmental stresses (Foyer et al., 2001). Higher GSH concentration, particularly GSSG/GSH ratios in Dong Dan 80 might be associated with low H_2_O_2_ concentrations. It might be assumed that maize plants depend on constitutive GSH to counteract the drought-related oxidative stress, where enhanced non-enzymatic functioning in Dong Dan 80 enhanced its ability to withstand the drought stress. Our results corroborate those of Zhang et al. (2013) who demonstrated that drought stress exacerbated the production and accumulation of non-enzymatic antioxidants in drought tolerant *Canna edulis.*

In summary, maize growth and yield responses were related to ROS production, osmolyte accumulation and activation of anti-oxidative defense system under drought conditions. Dong Dan 80 performed better with minimum yield losses and proved drought tolerant with enhanced osmolyte accumulation, and efficient enzymatic and non-enzymatic anti-oxidative defense systems even at SD stress conditions than Wan Dan 13 or Run Nong 35. In future, physiological and biochemical indices-based selection of drought tolerant germplasm may be helpful to develop novel drought-resistant genotypes that can be successfully cultivated in field conditions even at limited water supply.

## Author Contributions

SA and UA conducted experiment, MT, IK, SH, BS, and AZ assisted in data analyzing and manuscript write up, FA, IA, and MS assisted in write up and English improvement. LW supervised the study.

## Conflict of Interest Statement

The authors declare that the research was conducted in the absence of any commercial or financial relationships that could be construed as a potential conflict of interest.

## Abbreviations

APX: ascorbate peroxidase
AsA: total ascorbate
BY: biological yield
CAT: catalase
DAP: days after planting
DHA: de hydro ascorbate
DHAR: dehydroascorbate reductase
FC: field capacity
FP: free proline
GSH: reduced glutathione
GSSG: oxidized glutathione
GY: grain yield
LD: low drought
MDHAR: monodehydroascorbate reductase
MRD: moderate drought
POD: peroxidase
SD: severe drought
SOD: superoxide dismutase
SP: soluble protein
SS: Soluble sugars
SWD: shoot dry weight
TC: total carbohydrates
TFA: total free amino acids
TPC: total phenolic contents
